# Differential regulation of the Saccharomyces cerevisiae iron regulon by the nonsense- mediated mRNA decay pathway under iron scarcity

**DOI:** 10.21203/rs.3.rs-11245789/v1

**Published:** 2026-10-16

**Authors:** Jacqueline Carroll, Jana Chao, Bessie W. Kebaara

## Abstract

The nonsense-mediated mRNA decay (NMD) pathway is a conserved eukaryotic pathway that targets mRNAs prematurely terminating translation. Some endogenous, non-erroneous mature mRNAs are regulated by the NMD pathway, depending on the cellular context and extracellular stimuli. Prior studies have implicated NMD in maintaining cellular iron homeostasis, though to what extent is unknown. In this study, the effects of NMD on iron scarcity were examined in wild-type and NMD mutant *(upf1Δ)* strains by examining growth, iron content via inductively coupled plasma mass spectrometry (ICP-MS), transcriptome-wide analysis of potential NMD targets, and further validation of identified potential NMD targets. We found that cells with a nonfunctional NMD ( *upf1∆* ) pathway have impaired growth in iron scarcity compared to wild-type cells. The NMD mutant strain accumulated higher levels of intracellular iron than the wild-type in both normal growth conditions and iron deplete conditions. Transcriptional activators of the iron regulon, *AFT1* and *AFT2* were observed to be NMD targets depending on growth condition and iron availability. Downstream targets of *AFT1/2* include the ferric-cupric reductases *FRE1, FRE2* , *FRE3* , and *FRE4* ; these were found to produce multiple mRNA isoforms that were also subject to NMD-mediated regulation under iron sufficiency and scarcity. Growth conditions of the cells impact NMD-mediated expression of *FRE1-4* mRNAs and elucidates a temporal relationship of NMD-mediated regulation dependent on the duration of iron scarcity.

## Introduction

The Nonsense-mediated mRNA decay (NMD) pathway is a highly conserved translation-dependent mRNA degradation pathway within eukaryotic organisms (Amrani, Ganesan et al. 2004, Amrani, Sachs et al. 2006, Celik, Baker et al. 2017, Nickless, Bailis et al. 2017, Andjus, Morillon et al. 2021). The NMD pathway was named after the function for which it was initially discovered in *Saccharomyces cerevisiae*, serving as a mode of mRNA surveillance, and targeting mRNAs containing premature termination codons (PTCs) for degradation (Amrani, Ganesan et al. 2004, Amrani, Sachs et al. 2006, Celik, Baker et al. 2017, Nickless, Bailis et al. 2017, Andjus, Morillon et al. 2021). However, the NMD pathway has also been shown to have additional functions including regulating gene expression by targeting non-erroneous, fully functional, naturally occurring mRNAs (Amrani, Ganesan et al. 2004, Amrani, Sachs et al. 2006, Peccarelli and Kebaara 2014, Celik, Baker et al. 2017, Andjus, Morillon et al. 2021). Degradation of natural mRNAs by NMD has been observed in multiple organisms including *S. cerevisiae*, *C. elegans*, *Drosophila*, mice, and humans (Nickless, Bailis et al. 2017). In yeast, the three core factors essential for NMD to function are encoded by *UPF1*, *UPF2*, and *UPF3*, and the deletion of any one of these core factors renders the pathway nonfunctional (Leeds, Peltz et al. 1991, Atkin, Schenkman et al. 1997, Amrani, Ganesan et al. 2004, Celik, Baker et al. 2017).

Though the mechanisms of NMD-mediated regulation of natural mRNAs are not fully understood, varying factors have been shown to affect transcript sensitivity to NMD, such as features of the transcripts. These mRNA features include atypically long 3'Untranslated regions (UTRs), translated upstream open reading frames (uORFs), ribosomal frameshifting, and leaky ribosomal scanning resulting in out-of-frame translation initiation (Gaba, Jacobson et al. 2005, Guan, Zheng et al. 2006, Kebaara and Atkin 2009, Peccarelli and Kebaara 2014). Additionally, transcripts can be differentially regulated by NMD due to changes in cellular context and environmental conditions such as extracellular concentration of metal ions (Peccarelli and Kebaara 2014, Peccarelli, Scott et al. 2016, Peccarelli, Scott et al. 2019, Wong, Lam et al. 2021, Zhang and Kebaara 2022, Zhang, Olaniyan et al. 2025).

A pervasive regulatory role of NMD is the regulation of gene expression in bio-metal homeostasis (Peccarelli, Scott et al. 2016, Peccarelli, Scott et al. 2019, Zhang and Kebaara 2022, Zhang, Olaniyan et al. 2025). mRNAs involved in bio-metal homeostasis and regulated by NMD include the main magnesium transporter, *ALR1* (Johansson and Jacobson 2010). Further, transcripts responsible for the inhibition of genes implicated in zinc and iron homeostasis are also regulated by NMD (Toesca, Nery et al. 2011). Additionally, we found that NMD regulates multiple transcripts encoding proteins that play roles in copper homeostasis (Peccarelli, Scott et al. 2016, Zhang, Olaniyan et al. 2025). Furthermore, yeast with a functional NMD pathway experience impaired growth when subjected to toxic levels of environmental copper (Deliz-Aguirre, Atkin et al. 2011, Wang, Okonkwo et al. 2013, Wong, Lam et al. 2021). In yeast, copper and iron homeostasis are linked and partially mediated by NMD (Wang, Okonkwo et al. 2013, Peccarelli, Scott et al. 2016, Wong, Lam et al. 2021). Copper is a cofactor that is required for iron uptake (Dancis 1998), as well as mitochondrial oxidative phosphorylation. Iron is also an important cofactor for metabolic processes, including respiration, DNA synthesis, and translation. Though copper and iron are essential for normal cellular function, excess amounts of either metal is toxic and can lead to cellular damage. Thus, maintaining proper copper and iron homeostasis is imperative to organism health.

*S. cerevisiae* has been used as a model to study iron, and previous studies have shown that iron homeostasis is regulated at both the transcriptional, post-transcriptional and post-translational levels (Outten and Albetel 2013, Martins, Costa et al. 2018, Ramos-Alonso, Romero et al. 2020). Under low iron conditions, yeast cells will experience a shift in gene expression to promote iron intake and shift internal metabolic processes to efficiently utilize iron stores for essential cellular processes. The collection of genes involved in this metabolic shift is referred to as the iron regulon. There are two major transcription factors that activate the iron starvation response. The genes that encode these factors are *AFT1* and its paralogue, *AFT2* (Rutherford, Jaron et al. 2003, Miethke and Marahiel 2007, Outten and Albetel 2013, Martinez-Pastor, Perea-Garcia et al. 2017). Despite being constitutively expressed under normal growth conditions, Aft1p does not activate the iron starvation response, as it remains in the cytoplasm. Under low iron conditions, Aft1/2p localizes to the nucleus to activate genes necessary for increasing iron uptake from the environment and shifting cellular processes to mobilize internal iron stores (Bird 2008, Martins, Costa et al. 2018, Ramos-Alonso, Romero et al. 2020). There is notable overlap between the individual collections of downstream targets of Aft1p and Aft2p (Blaiseau, Lesuisse et al. 2001, Rutherford, Jaron et al. 2001). Aft2p can continue regulating the expression of genes involved in maintaining iron homeostasis in the absence of Aft1p (Blaiseau, Lesuisse et al. 2001, Rutherford, Jaron et al. 2001), however, it has also been observed that the downstream targets of Aft1p are primarily involved in iron acquisition while downstream targets of Aft2p facilitate a metabolic shift within the cell for optimizing cellular iron stores (Rutherford, Jaron et al. 2001, Courel, Lallet et al. 2005).

Three pathways import iron across the cell membrane in yeast; these pathways include 1) the nonreductive $Fe^{3+}$ pathway, facilitated by the ARN family of iron siderophore transporters, 2) the reductive $Fe^{3+}$ pathway facilitated by members of the FRE family of ferric-cupric reductases in conjunction with the oxidoreductase Fet3p and high-affinity iron permease Ftr1p, and 3) the $Fe^{2+}$ import pathway facilitated by divalent metal ion transporter Smf1p and low-affinity iron transporter Fet4p (Lindahl 2019). Studies have established that *FRE1* is regulated by both Aft1/2p, while *FRE2-6* are all targets of only Aft1p. Additionally, *ARN1, ARN2 (TAF1), ARN3 (SIT1)*, and *ARN4 (ENB1)* are all targets of only Aft1p (Rutherford, Jaron et al. 2003). While the divalent metal ion transporter, *SMF1* is not a target of the Aft1/2p iron regulon, it does play an important role in iron homeostasis; it has been demonstrated that in addition to playing a role in other metal ion acquisitions, Smf1p can also facilitate iron uptake in a compensatory manner when primary iron uptake pathways are dysfunctional. In yeast cells lacking both multicopper oxidases Fet3p and Fet4p, the expression of *SMF1* resulted in a rise of iron uptake by the cell (Cohen, Nelson et al. 2000).

Siderophores are low-molecular weight molecules secreted by unicellular organisms to chelate ferric ($Fe^{3+}$) iron in preparation for cellular uptake (Cain and Smith 2021). *S. cerevisiae* is unable to produce siderophores, however yeast can employ an "opportunistic strategy" of ferric ($Fe^{3+}$) iron uptake by taking in siderophores that are produced by other microorganisms (Lesuisse, Blaiseau et al. 2001, Miethke and Marahiel 2007). The ARN family of genes encode for membrane receptors that facilitate the uptake of chelated ferric ($Fe^{3+}$) iron siderophores into yeast cells (Yun, Tiedeman et al. 2000). However, the uptake of siderophore-bound iron is also dependent on the reductive activity of the FRE family of plasma membrane-bound ferric-cupric reductases (FRE1-4p) to reduce insoluble ferric ($Fe^{3+}$) iron to a soluble ferrous ($Fe^{2+}$) form (Dancis 1998, Yun, Bauler et al. 2001, Cain and Smith 2021).

Previous genome-wide studies have primarily identified NMD targets in *S. cerevisiae* cells grown in rich media (Yeast Extract Peptone and Dextrose (YEPD) or defined synthetic minimal media (He, Li et al. 2003, Guan, Zheng et al. 2006, Johansson, He et al. 2007, Celik, Baker et al. 2017). Here, we investigated NMD-mediated regulation in iron deficient contexts to assess the similarities and differences in the transcriptional response under normal growth conditions and iron-deficiency.

We first examined how NMD modulated iron scarcity by measuring growth and iron content of yeast cells grown under CM and iron-deficient conditions. We found the yeast cells with a non-functional NMD pathway (*upf1Δ*) had impaired growth relative to the wild-type strain under iron deficiency. Furthermore, the NMD mutant accumulated higher levels of intracellular iron relative to the wild-type strain in both deplete and replete conditions. Analysis of prior RNA-Seq data of the 139 genes identified as part of the ironome in *S. cerevisiae* (Lindahl 2019), found that 25 of these genes encode mRNAs that are potential NMD targets under normal growth conditions. Further analysis of wild-type and NMD mutant (*upf1Δ)* yeast cells grown in normal growth conditions and hourly timepoints up to 3 hours of iron-deficiency showed changes in expression of genes involved in response to iron starvation. Differential expression of the transcriptional activators of the iron regulon may be reflected in expression levels of downstream targets. Therefore, we examined the levels of select mRNAs of the iron regulon. We examined the levels of plasma membrane ferric-cupric reductases *FRE1*, *FRE2*, and *FRE3*, a category of highly represented genes upregulated in NMD mutants under normal growth conditions, and we previously investigated the role NMD-mediated regulation of *FRE1* and *FRE2* plays in copper metabolism (Peccarelli, Scott et al. 2019). The *FRE* family of genes are activated by Aft1p/Aft2p under low iron conditions (Blaiseau, Lesuisse et al. 2001, Rutherford, Jaron et al. 2001, Courel, Lallet et al. 2005, Rutherford, Ojeda et al. 2005, Lindahl 2019).

### Materials and Methods

## Growth of wild-type and NMD mutant yeast cells in low iron:

Wild-type and NMD mutant (*upf1Δ*) yeast strains were grown in Complete Minimal (CM) media to mid-log phase overnight (Wente, Rout et al. 1992, Kebaara, Nazarenus et al. 2003)(**Supplementary Table 1**). Triplicate CM growth curves were done by diluting overnight cultures with CM to an $OD_{600}$ of 0.1 and taking subsequent measurements every 2 hours for 12 hours. For low iron growth curves, overnight cultures were centrifuged and rinsed twice with sterile deionized $H_2O$, before resuspending the cells in 10 ml of low iron media and diluting back down to an $OD_{600}$ of 0.1 for measurements every 2 hours for 12 hours. For plates, triplicate drop tests were also prepared from mid-log phase cultures; four- tenfold serial dilutions ($10^0$, $10^{-1}$, $10^{-2}$, $10^{-3}$) were prepared and subsequently spotted onto CM and low iron (CM + 100 μM metal chelator Bathophenanthrolinedisulfonic acid, abbreviated BPS) agar plates. BPS is a chelator of ferrous ions generating $[Fe^{II}(BPS)_3]^{4-}$(Holmes-Hampton, Jhurry et al. 2013). Plates were incubated at 30°C for 6 days. Complementation of yeast strains with pRS315 (empty vector) and pRS315UPF1 (Functional Upf1p) was accomplished via lithium acetate-mediated transformation into both wild-type and NMD mutants (Gietz and Woods 2002), (Sikorski and Hieter 1989) (Atkin, Schenkman et al. 1997). Cell viability was assessed for CM, as well as 0, 1, 2, and 3 hours of iron deprivation by mixing 10 μl of undiluted culture with 10 μl of 0.05% methylene blue at the specified timepoints. Cells were incubated at room temperature for 5 minutes before spotting 5 μl onto a hemocytometer for counting.

### Measurement of cellular iron levels:

Cellular iron content was measured in triplicate via inductively coupled plasma mass spectrometry (ICP-MS). For Complete Minimal (CM, normal iron conditions), wild-type and NMD mutant yeast cells were grown to an $OD_{600}$ of 1.0 (1 X $10^7$ cells/ml) before 1 ml cell harvest. For measurement of high iron content cells were grown to an $OD_{600}$ of 1.0 (1 X $10^7$ cells/ml) in high iron (CM + 100 μM of Fe(III)Cl) media before 1 ml cell harvest. Low iron cells were grown to an $OD_{600}$ of 1.0 (1 X $10^7$ cells/ml) in Complete Minimal (CM) media before being rinsed twice with deionized $H_2O$ and resuspended in 25 ml of low iron (CM + 100 µM BPS). Low iron cells were equilibrated in low iron media for 3 hours before 1 ml cell harvest (Romero, García-Martínez et al. 2022). Harvested cells were washed three times with 1 mM EDTA to remove non-specifically bound metals. Cells were then sent for ICP-MS analysis. Analysis included digesting the cells with 70% nitric acid for 2 hours at 70°C before 20-fold dilution in deionized $H_2O$ before ICP-MS analysis. Unpaired t-tests were conducted to evaluate significant differences between wild-type and NMD mutants, and between conditions

### Growth of yeast cells for measurement of mRNA accumulation levels:

Wild-type and NMD mutant yeast strains were used for steady-state mRNA accumulation experiments (Kebaara, Nazarenus et al. 2003). mRNA accumulations were done using two protocols. In the first protocol, wild-type and NMD mutant cells were grown to saturation overnight. Subsequently, the cells were sub-cultured into CM or low iron media (CM + 100 µM BPS) and allowed to grow to mid-log phase before harvesting. In the second protocol the yeast cells were grown in Complete Minimal (CM) growth conditions to mid-log phase. Cells were then washed twice with deionized $H_2O$ before resuspension in 25mL low iron (CM + 100 µM BPS) media. After resuspension in low iron media, yeast cells were harvested at 0, 1, 2, and 3-hour intervals and immediately frozen for further processing (Romero, García-Martínez et al. 2022).

### RNA extraction from yeast and quantitative northern blotting

RNA extraction from yeast and quantitative northern blotting
Total RNA was extracted via the hot phenol method (Kohrer 1991, Leeds, Peltz et al. 1991). The RNA concentration was quantified using the Nanodrop, and the RNA was diluted to 1 ug/µl. Equivalent concentrations of total RNA were run on a 1.0% agarose-formaldehyde gel. The RNA was then transferred to a GeneScreen Plus® (PerkinElmer, Boston, MA) nitrocellulose membrane. Transfers were done using the capillary blot transfer protocol from the NorthernMax™ Complete Northern Blotting Kit (Ambion, Inc., Austin, TX). After transfer, the RNA was cross-linked to the membrane via the UVC500 UV Cross-Linker (Hoefer, Holliston, MA).

Northern blots were hybridized with specific oligo-labeled DNA probes sequentially. All transcripts were detected by probes specific to their Open Reading Frame (ORF). Primers flanking the Open Reading Frame (ORF) of each gene of interest that were used to generate the probe fragments used in this study (**Supplementary Table 2**). The oligo-labeled probes were prepared with [a-$^{32}$P] dCTP using the RadPrime DNA Labeling System (Invitrogen, Carlsbad, CA). The northern blots were then probed with the oligo labelled probes. After probing, the blots were washed and wrapped individually in Saran Wrap before exposure to a phosphorimaging screen. The blots were then visualized using the Typhoon Phosphorimager (Cytiva life sciences). RNA isoform sizes were estimated by comparing their migration distances on the northern blot to those of the RNA ladder.

### Quantitative analysis:

Northern Blots were done in triplicate and quantified utilizing ImageQuant software (Cytiva life Sciences). RNA Band intensity was measured with ImageQuant software and background signal was subtracted. Bands were then normalized to *SCR1* loading control. Normalized values were used to calculate fold change (NMD mutant/wild-type). One-tailed, t-tests were conducted in excel evaluating whether mean fold changes were significantly different from a value of 1, where wild-type expression is equivalent to a fold-change of 1. Significance is denoted as $p < 0.05$ = *, $p < 0.01$ = **).

## Results

**NMD mutant ( upf1 Δ) strains have a slight growth impairment in low iron conditions relative to the wild-type strains.**

To distinguish the tolerance of the wild-type and NMD mutant (*upf1Δ)* yeast strains to iron-deficient conditions, the strains were grown in Complete Minimal media which contained 0.46 (± 0.13) μM iron (we regarded this level as normal iron), and low iron media (CM containing 100 μM of the iron chelator bathophenanthrolinedisulfonic acid (BPS)) on plates and in liquid media.

Both strains exhibited impaired growth under iron deficient conditions relative to CM (Fig. 1A, 1B). Notably, the NMD mutant strains had reduced growth relative to the wild-type strain in iron-deficient conditions (Fig. 1B, 1E**)**. Complementation of the NMD mutant strain with a functional Upf1 restored growth of the NMD mutant under low iron conditions in the plate assay, resulting in equivalent growth with the wild-type (Fig. 1D, highlighted by a red rectangle).

Growth curves in liquid media demonstrated a modest growth defect of the NMD mutant relative to the wild-type in CM that became more pronounced under low iron conditions (Fig. 1E), suggesting that iron limitation exacerbates the growth defect of the NMD mutant. Comparison of the calculated average doubling times via Welch's t-test of the wild-type and NMD mutant yeast strains under low iron (2.87 hours and 3.26) hours, respectively was found to be significant ($p = 0.031$). Complementation with the leucine vector (pRS315) did not substantially alter the growth phenotypes of either the wild-type or NMD mutant strain in media lacking leucine or low iron lacking leucine (Fig. 1F). Complementation with functional Upf1p resulted in comparable growth of the wild-type and NMD mutant strains in media lacking leucine or low iron minus leucine as no statistically significant difference was found between growth rates of either strain in either condition. This suggests that restoration of Upf1 can rescue the growth defect associated with the loss of NMD under these conditions. Consistent with the growth curves, supplementary analysis of the area under the curve (AUC) confirmed the reduced overall growth of the NMD mutant relative to the wild-type, particularly under low iron conditions, and supported the partial rescue of the mutant growth phenotype by complementation of functional Upf1 in the NMD mutant (**Supplementary Fig. 1A, 1B**).

Additionally, cell viability of the wild-type and NMD mutant *(upf1Δ )* yeast strains were assessed in CM, and 0, 1, 2, and 3 hours of iron deprivation (Fig. 1G). Cell viability remained consistently high (> 98%) throughout the 3-hour iron deprivation time course in both wild-type and NMD mutant cells, indicating that short-term iron deprivation did not substantially impair cell survival. Although analysis revealed a significant effect of time in low iron media on cell viability ($p = 0.007$), the observed changes in viability were small (< 1%). Post-hoc Sidak's multiple comparisons testing revealed a modest transient decrease in viability between 0 and 1 hour of iron deprivation in wild-type cells ($p = 0.041$, indicated by the asterisk). Neither genotype nor time x genotype interaction were found to be significant, indicating that deletion of *UPF1* did not affect cell viability during the short-term iron starvation event. Together, these results suggest that *UPF1* and thus the NMD pathway is required for efficient adaptation to iron deprivation rather than maintenance of cell viability under short-term iron starvation.

**Intracellular iron levels vary when wild-type and NMD mutant (upf1Δ) yeast strains are grown under normal, low and high iron conditions.**

To quantify the total cellular iron content in wild-type and NMD (*upf1*Δ) mutants, the cells were grown in three conditions: CM (normal iron levels), low iron (CM + 100µM BPS) and high iron (CM + 100µM Fe(III)Cl) conditions. The wild-type strain had a cellular iron concentration of 3.5 (± 0.3) µM, while the NMD mutant (*upf1Δ)* yeast strain had a cellular iron concentration of 21 (± 5.0) µM in CM (Table 1, Fig. 2). The significant difference ($p = 0.049$) in cellular iron concentrations between the wild-type and NMD mutant suggests that NMD plays a regulatory role in genes involved in extracellular iron uptake under CM conditions. The wild-type and NMD mutant were examined in low iron conditions, the wild-type strain had a cellular iron concentration of 1.0 (± 0.01) µM, while the NMD *(upf1Δ)* mutant had a cellular iron concentration of 2.7 (± 0.8) µM (Table 1, Fig. 2). Although the difference between the wild-type and mutants was not statistically significant ($p = 0.058$), the iron content was trending towards higher cellular concentrations in the NMD mutant *(upf1Δ)* cells compared to the wild-type under low iron conditions. In high iron conditions, the wild-type strain was found to have a cellular iron concentration of 1176 (± 56) µM, while the NMD mutant had a cellular iron concentration of 867 (± 20) µM (Table 1, Fig. 2). This suggests that in high iron conditions some NMD targeted genes involved in iron uptake could be affected.

Table 1
Iron content measured using inductively coupled plasma mass spectrometry (ICP-MS) in wild-type (*UPF1*) and NMD mutant (*upf1Δ*) yeast cells. Intracellular iron content of the wild-type and NMD mutant yeast strains was measured via inductively coupled plasma mass spectrometry (ICP-MS in iron-sufficient conditions (CM), low iron conditions (CM + 100 μM BPS), and high iron conditions (CM + 100 μM Fe(III)Cl). Iron levels are reported in μM ± standard deviation (SD).

| Growth Media | Wild-type (*UPF1*) | NMD Mutant (*upf1Δ*) |
|---|---|---|
| **Complete Minimal** | 3.5 (± 0.3) μM | 21 (± 5.0) μM |
| **CM + 100 μM BPS*** | 1.0 (± 0.1) μM | 2.7 (± 0.8) μM |
| **CM + 100 μM Iron** | 1176 (± 56) μM | 867 (± 20) μM |
| *Bathophenanthrolinedisulfonic acid | | |

Bio-metal homeostatic mechanisms are linked. Changes in metal ion concentrations influence the balance of other metal ions. Thus, total intracellular levels of copper and zinc were also examined in the same conditions. Significant differences in the levels of intracellular copper were detected between the wild-type and NMD mutant (*upf1Δ*)yeast strains grown in low iron and high iron. However, no significant differences in intracellular copper levels were observed between wild-type and NMD mutants within their respective growth conditions (**Supplementary Fig. 2A, Supplementary Table 3**). No significant difference in intracellular zinc concentrations was observed between wild-type and NMD mutant (*upf1Δ)* cells exposed to low iron conditions compared to CM or high iron conditions (**Supplementary Fig. 2B, Supplementary Table 4**). However, there was significant difference in intracellular levels of zinc in both the wild-type and NMD mutant strains (*upf1Δ)* when comparing yeast strains grown in CM and low iron conditions. Significant differences in intracellular zinc levels were also found between the wild-type yeast strains grown in low iron and high iron conditions, as well as NMD mutant yeast strains grown in low iron and high iron conditions. Collectively, these findings suggest that reduced iron availability variably impacts intracellular copper and zinc concentrations, though disruption of NMD does not significantly alter the accumulation of these metal ions under the tested conditions.

## RNA-seq studies reveal potential NMD targets

Of the 139 genes identified as the ironome within *S. cerevisiae* in (Lindahl 2019), we found 25 of these genes encode mRNAs that have been identified as NMD targets under iron-sufficiency (Fig. 3, **Supplementary Table 5**). Among these regulated transcripts, *AFT2* was reported to associate with Upf1p, providing evidence of being a direct NMD target (Johansson, He et al. 2007). In contrast, information regarding potential NMD targeting of *AFT1* is lacking. This could indicate that *AFT1* is not regulated by NMD under normal growth conditions, or that the effects of NMD-mediated regulation on *AFT1* are far more subtle than its paralogue and has not yet been reported. Given the strong evidence for *AFT2* as a potential NMD substrate, it is important to examine if *AFT1* and *AFT2* are NMD targets under inducing low iron conditions. Furthermore, downstream targets of Aft1/Aft2p may be NMD-sensitive, indirectly modulated by NMD through regulation of *AFT1/AFT2*, or they may be direct NMD targets themselves. However, there is significant overlap between Aft1p and Aft2p targets (Rutherford, Jaron et al. 2001, Rutherford, Jaron et al. 2003, Courel, Lallet et al. 2005, Lindahl 2019, Ramos-Alonso, Romero et al. 2020).

Examination of RNA-seq data reported by (Zhang, Olaniyan et al. 2025) comparing NMD wild-type (*UPF1*) and mutant (*upf1Δ*) yeast strains in Complete Minimal (CM) conditions identified 14 genes related to iron homeostasis that were upregulated in the NMD mutant (Table 2). The siderophore transporters *ARN1*, *ARN2*, and *ARN4* showed the largest increases in *upf1*Δ mutants. Several members of the FRE family of metalloreductases also exhibited higher transcript abundance in the NMD mutant, including those localized to the plasma membrane, *FRE2*, *FRE3*, and *FRE4*. The mitochondrial and vacuolar reductases, *FRE5* and *FRE6* respectively, showed a more moderate fold change. Interestingly, *FRE7*, whose expression is induced under low copper conditions also accumulated in the NMD mutant (Nevitt, Ohrvik et al. 2012).

Table 2
RNA-seq fold changes of the iron-related genes that are upregulated in the NMD mutant (*upf1Δ*) compared to the wild-type (*UPF1*) in CM conditions (Zhang, Olaniyan et al. 2025).

| Number | Name | Gene | Function | RNA-Seq<br><br>Fold Change in CM (*upf1Δ / UPF1)* |
|---|---|---|---|---|
| 1 | YHL040C | *ARN1* | Transporter for ferrichrome iron chelate | 2.63 (± 0.2) |
| 2 | YHL047C | *ARN2* | Uptake of iron triacetylfusarinine | 2.67 (± 0.4) |
| 3 | YOL158C | *ARN4* | Endosomal ferric enterobactin transporter | 3.10 (± 0.4) |
| 4 | YKL220C | *FRE2* | Metalloreductase | 2.90 (± 0.4) |
| 5 | YOR381W | *FRE3* | Metalloreductase | 3.05 (± 0.5) |
| 6 | YNR060W | *FRE4* | Metalloreductase | 2.70 (± 0.2) |
| 7 | YOR384W | *FRE5* | Mitochondrial Metalloreductase | 2.32 (± 0.3) |
| 8 | YLL051C | *FRE6* | Vacuolar Metalloreductase | 1.76 (± 0.4) |
| 9 | YOL152W | *FRE7* | Metalloreductase | 2.65 (± 0.4) |
| 10 | YIL003W | *CFD1* | Cytosolic iron-sulfur cluster assembly | 1.98 (± 0.4) |
| 11 | YNL260C | *LTO1* | Required for insertion of Fe/S cluster into apo-Rli1p | 1.98 (± 0.2) |
| 12 | YBL071W-A | *DPH3* | Required for peptidyl-diphthamide formation from peptidyl-histidine and wobble uridine tRNA modification | 2.60 (± 0.2) |
| 13 | YDR151C | *CTH1* | Translational repressor in low iron conditions | 2.80 (± 0.3) |
| 14 | YOL043C | *NTG2* | Involved in base excision repair | 1.77 (± 0.2) |
| Figures legends | | | | |

Two genes involved in iron–sulfur cluster assembly were also elevated. *CFD1* encodes a highly conserved iron-sulfur cluster binding protein involved in cytoplasmic iron-sulfur protein assembly and accumulated in the NMD mutant. *LTO1*, also required for the insertion of iron-sulfur clusters for ribosome-associated protein maturation accumulated to similar levels (Paul, Mühlenhoff et al. 2015). Additionally, accumulation of *DPH3*, which is required for dipthamide biosynthesis on translation elongation factor 2 (eEF2) (Kolaj-Robin, McEwen et al. 2015), *CTH1*, a regulator of iron-utilizing mRNAs, and *NTG2*, which is involved in redox-linked DNA repair, all accumulated to higher levels in the NMD mutant. Collectively, these findings suggest NMD-mediated regulation of genes associated with iron uptake from the environment and Fe–S cluster biogenesis, highlighting a broad influence of NMD on iron homeostasis pathways under normal growth conditions.

**NMD -mediated regulation of AFT1, AFT2 and downstream targets is dependent on iron levels.**

To elucidate the extent to which NMD regulates the transcriptional activators of the iron regulon under low iron inducing conditions, we grew wild-type (*UPF1)* and NMD mutant *(upf1Δ)* strains in CM and low iron media to mid-log phase, extracted RNA, and measured the steady-state accumulation levels of *AFT1* and *AFT2* transcripts. Under these conditions, *AFT1* encoded one predominant mRNA isoform that accumulated 1.68 (± 0.41)-fold in the NMD mutant compared to the wild-type under normal growth conditions (**Supplementary Fig. 3**). In low iron and inducing conditions for *AFT1*, the transcript accumulated to higher levels in the wild-type strain compared to the NMD mutant (fold change of 1.01 ± 0.21, $p = 0.477$).

Examination of *AFT2* mRNA revealed a single predominant isoform that accumulated to higher levels in the NMD mutant relative to the wild-type, 1.77 (± 0.65, $p = 0.055$) fold in CM. Under inducing low iron conditions, the fold change of *AFT2* was 0.91 (± 0.28, $p = 0.359$), indicating modified regulation by NMD. *AFT2* mRNA was previously reported as a direct NMD substrate because it directly associates with Upf1p, under normal growth conditions (Johansson, He et al. 2007). These findings suggest that *AFT2* is an NMD target under non-inducing conditions. They also demonstrate that NMD regulates activators of the low-iron regulon differently depending on iron availability.

The ferric cupric reductase transcripts *FRE1-3*, responded to NMD and iron deprivation in different ways. *FRE1* produced two mRNA isoforms under low iron conditions both of which do not accumulate to higher levels in the NMD mutant relative to the wild-type and consistent with our previous reports that *FRE1* is not an NMD target in low iron conditions (Peccarelli, Scott et al. 2019). *FRE2* generated three isoforms in low iron conditions that were undetectable in the non-inducing CM condition if detected on the same northern blot as low iron (inducing conditions). *FRE2* mRNA isoforms are detectable in CM if they are not on the same northern blot as low iron conditions (Peccarelli, Scott et al. 2019). The most predominant isoform escapes regulation in low iron conditions. The third member of the *FRE* family, *FRE3*, has been previously reported to be a direct NMD target under normal growth conditions and found to be downregulated upon NMD-reactivation (Guan, Zheng et al. 2006, Johansson, He et al. 2007). In agreement with these prior reports of NMD sensitivity, *FRE3* produced mRNA isoforms that accumulate to higher levels in the NMD mutant compared to the wild-type in CM (**Supplementary Fig. 3A)**. Under low iron conditions, the predominant *FRE3* isoform which was undetectable in CM accumulates to 1.93 (± 0.5, $p = 0.017$)-fold higher levels in the NMD mutant compared to the wild-type.

**Temporal regulation of AFT1, AFT2 and FRE1-4 mRNAs by NMD over three hours of iron starvation**

The above analysis of mRNA steady-state accumulations comparing CM and low iron conditions were performed under conditions of iron starvation, as the yeast cells were grown to mid-log phase ($OD_{600}$ 0.5–0.7) in low iron media. Under prolonged low iron conditions, yeast cells have impaired growth (Fig. 1B, 1E). This selective pressure could have contributed to adaptation to these conditions, leading to altered regulation of mRNAs by NMD and failure to detect transcripts that are sensitive to NMD during initial stages of iron deprivation. To examine temporal regulation of the mRNAs by NMD we grew wildtype and NMD mutant (*upf1Δ*) strains in CM to mid-log phase, washed the cells twice with deionized water, before resuspending the cells in low iron media. Subsequently, cells were sampled hourly for three hours.

At 0 hours of iron deprivation, *AFT1* encoded the same predominant, long mRNA isoform (~ 4.3 kb) that was detected in CM and low iron conditions (**Supplementary Fig. 3A).** This *AFT1* mRNA isoform did not accumulate to higher levels in the NMD mutant (*upf1Δ*) compared to the wild-type strain at 0 and 1 hour of iron deprivation **(**Fig. 4A, 4B**)**. Thus, NMD targeting was undetectable at the beginning of an iron starvation event. However, accumulation of this major isoform was observed at 2 hours of iron deprivation (fold-change of 1.42 ± 0.1, p = 0.012) and appeared to increase slightly at 3 hours of iron deprivation (fold-change of 1.56 ± 0.2, p = 0.024) (**Supplementary Table 6**).

*AFT2* also encodes multiple mRNA isoforms with one long, predominant isoform (~ 2.8 kb) that was also the major isoform detected in CM (**Supplementary Fig. 3A)**. This *AFT2* mRNA isoform accumulated to higher levels in the NMD mutant (*upf1Δ*) at 0, 1, 2, or 3 hours of iron deprivation relative to the wild-type, indicating the potential for low level NMD sensitivity throughout the iron starvation event **(**Fig. 4A, 4C**)**. When cells were exposed to prolonged low iron conditions *AFT2* mRNA appeared insensitive to NMD (**Supplementary Table 6**, Fig. 4B). However, statistical analysis of the fold-changes of *AFT2* demonstrated no significant difference of the means from a fold-change of 1 at any time point (**Supplementary Table 6**).

Examination of *FRE1-FRE4* mRNAs, which are downstream targets of Aft1p and Aft2p, revealed different results relative to yeast cells grown to mid-log phase in low iron (**Supplementary Fig. 3**). *FRE1* mRNA isoforms accumulated immediately after switching cells to iron deprivation conditions compared to CM (Fig. 5A, 5B **Supplementary Table 6**). The accumulation of the predominant ~ 2.3 kb *FRE1* isoform increased at the 2-hour timepoint (fold change of 2.84 ± 0.4, p = 0.009), before leveling off at 3 hours of iron deprivation (**Supplementary Table 6**).

*FRE2* was identified as an NMD target in CM conditions (Peccarelli, Scott et al. 2019). Four *FRE2* isoforms of varying lengths were detected with one major isoform appearing in both growth conditions. At 0 hours of iron deprivation, while nearly undetectable, the predominant *FRE2* mRNA isoform (medium) accumulated to higher abundance in the NMD mutant (*upf1Δ*) relative to the wild-type strain (Fig. 5A, 5C, **Supplementary Table 6**). Additionally, under inducing (low iron) conditions, accumulation was maintained during the 2 and 3 hours timepoints of iron starvation to significant degrees (fold-changes of 5.23 ± 2.0, p = 0.034; and 4.89 ± 1.3 p = 0.017, respectively) indicating consistent regulation of *FRE2* by NMD within iron deprivation conditions (**Supplementary Table 6**).

*FRE3* has also been previously reported to be regulated by NMD under normal growth conditions and identified as a direct NMD target (Guan, Zheng et al. 2006, Johansson, He et al. 2007). Three *FRE3* mRNA isoforms accumulated to higher levels in the NMD mutant (*upf1Δ*) compared to the wild-type strain while adapting to low iron conditions (Fig. 5A, **Supplementary Table 6**). The most predominant *FRE3* isoform (short) accumulated to much higher levels in the NMD mutant (*upf1Δ*) relative to the wild-type at 0 hours of iron deprivation (**Supplementary Table 6).** The magnitude of accumulation increased at each subsequent timepoint, with fold changes of 6.53 ± 0.6 (p = 0.002) at 1 hour, 10.58 ± 1.5 (p = 0.004) at 2 hours, and 9.95 ± 4.0 (p = 0.092) at 3 hours of iron deprivation, indicating a trend of increased NMD sensitivity over the duration of iron starvation (**Supplementary Table 6**). In summary, *FRE3* mRNAs accumulate to higher levels in the NMD mutants relative to the wild-type strains when cells are adapting to low iron conditions for a short time.

We also examined *FRE4* because it is the fourth ferric-cupric reductase located in the plasma membrane. Two isoforms of *FRE4* were detected on northern blots when wild-type and NMD mutants were adapting to low iron conditions (Fig. 5A, 5E, **Supplementary Table 6**). The predominant (short) isoform appeared to consistently accumulate to higher levels in the NMD mutant as compared to the wild-type strain within the duration of the iron starvation event, with fold changes of 4.11 ± 1.4 (p = 0.029) at 0 hours, 8.07 ± 5.4 (p = 0.075) at 1 hour, 4.16 ± 1.4 (p = 0.030) at 2 hours, and 3.05 ± 0.9 (p = 0.030) at 3 hours of iron deprivation (**Supplementary Table 6**). This indicates sustained regulation of *FRE4* mRNA by NMD under iron starvation conditions.

*FTH1* mRNA encodes a high affinity vacuolar iron transporter whose expression is regulated by iron as a target of Aft1p (Rutherford, Jaron et al. 2001, Courel, Lallet et al. 2005). RNA-seq data analysis did not reveal any accumulation of *FTH1* transcript above a fold-change threshold of 1.5. Northern blot analysis showed low level accumulation of *FTH1* mRNA transcript over the 3-hour iron starvation analysis which correlates with that of *AFT1* (Fig. 4A, **Supplementary Table 6**).

### NMD functionality upon iron starvation

Although iron deficiency is known to repress translation, a 3-hour iron starvation event is not sufficient to induce strong global repression of translation (Romero, Ramos-Alonso et al. 2020). Because iron serves as a cofactor for proteins in several steps of translation, studies on how low iron affects translation in *S. cerevisiae* have been done and found that 3 hours iron starvation does not significantly impact translation. Barlit et al, 2024, demonstrated that at 3 hours of iron deprivation transcriptional changes begin but translational repression is moderate (Barlit, Romero et al. 2024). At 6 hours of iron deprivation, they observed a strong translational inhibition. Romero et al 2020 observed that mRNA translation slightly decreased during a short-term (3 hours) exposure to iron starvation (Romero, Ramos-Alonso et al. 2020).

*CYH2* mRNA encodes a ribosomal protein L28. The *CYH2* pre-mRNA is inefficiently spliced and transported to the cytoplasm where it is degraded by NMD due to a stop codon in the intron (He, Peltz et al. 1993). *CYH2* mRNA is NMD-insensitive. Therefore, the ratio of *CYH2* pre-mRNA to mature mRNA provides an estimation of NMD functionality.

To assess NMD functionality, we evaluated the *CYH2* pre-mRNA to mRNA ratio. When cells were grown to mid-log phase in low iron conditions, the ratio of the *CYH2* pre-mRNA to mRNA was (0.033 (± 0.03)) in the wild-type strain indicative of a functional NMD pathway. In the NMD deficient strain the ratio was (0.52 (± 0.06)) indicating that there was an increased expression of the pre-mRNA, (**Supplementary Fig. 3A).** We also detected reduced expression of both the *CYH2* pre-mRNA and mRNA after 2- and 3-hours exposure to low iron (Fig. 4A, 4D). In the wild-type strain, this ratio was low at 0 hours (0.038 (± 0.01)) and 1 hour (0.05 (± 0.01)) of iron deficiency, consistent with efficient degradation of the NMD-sensitive *CYH2* pre-mRNA (Fig. 4D). However, the ratio of *CYH2* pre-mRNA to mRNA increased at hours 2 and 3 (Fig. 4D) suggesting a change in NMD efficiency or increased accumulation of pre-mRNA levels as the iron starvation event progressed. As NMD is a translation-dependent pathway, reduced translation could diminish NMD efficiency, leading to the modest accumulation of *CYH2* pre-mRNA in wild-type cells at later time points. In contrast, the NMD-deficient mutant exhibited elevated ratios throughout the experiment, 1.29 (± 0.13) at hour 0, followed by 0.65 (± 0.18), 0.89 (± 0.29), and 1.54 (± 0.61) at hours 1, 2, and 3 respectively, as expected for an NMD-deficient strain (Fig. 4D**)**. Thus, the progressive increase in the *CYH2* pre-mRNA to mature mRNA ratio in wild-type cells suggests that NMD is functional during iron starvation.

## Discussion

Here we provide multiple lines of evidence showing that NMD plays a role in iron metabolism in *S. cerevisiae*. First, NMD mutants have impaired growth under low iron and accumulate higher levels of cellular iron in CM and low iron conditions compared to the wild-type strain. Iron concentrations were measured using ICP-MS which does not determine the species of iron. However, accumulation of iron in the NMD mutant provides support not only for increased iron uptake systems but also iron trafficking systems due to differential mRNA regulation by NMD in response to iron deprivation. Under 100 µM iron conditions, we found that the wild-type strain has a higher cellular iron content relative to the NMD mutant strain (Fig. 2). As the iron regulon is an iron deprivation response and would not be activated in conditions of excess iron, the observed difference in cellular iron content between the wild-type and NMD mutant strains suggests that NMD may also regulate the opposite aspect of iron homeostasis. Additional investigation is warranted to explore the potential regulatory role of NMD on mRNAs involved in the iron toxicity response.

Perturbations in metal ion concentrations are known to influence the balance of others, such as cobalt, copper, zinc, and manganese (Philpott and Protchenko 2008). Low copper levels can impose a secondary limitation on iron acquisition, as the multicopper oxidase Fet3p, which complexes with the permease Ftr1p to form the high-affinity iron uptake system, requires copper to function (Stearman, Yuan et al. 1996, Dancis 1998). Insufficient copper compromises the complex's ability to utilize the ferrous ions generated by Fre1p and Fre2p ferric reductases, leading to reduced iron uptake and transcriptional mimicry of an iron starvation response. Analysis of our ICP-MS data demonstrated comparable copper levels between the wild-type and NMD mutant yeast strains in CM and low iron conditions (**Supplementary Fig. 2A**).

ICP-MS analysis also revealed that cellular levels of zinc were much higher in both the NMD mutant (*upf1Δ*) and wild-type strain under low iron conditions compared to either CM or 100 μM iron conditions (**Supplementary Fig. 2B, Supplementary Table 4**), highlighting the interconnected nature of metal ion homeostasis. Consistent with this, several genes involved in zinc uptake, including *ZRT1*, *FET4*, and *ZPS1*, have been reported as NMD targets under normal growth conditions (**Supplementary Table 7**). Notably, reduced levels of cellular iron and elevated levels of cellular zinc have been observed in wild-type and NMD mutant (*upf1Δ*) yeast strains under low copper conditions, (Zhang, Olaniyan et al. 2025). The low affinity metal ion transporter, Fet4p is regulated by both Aft1p and Zap1p, the transcriptional activator under zinc deficiency, and is known to transport zinc in addition to iron (Zhao, Cao et al. 2020). These observations suggest that iron starvation may indirectly promote zinc influx via transporters with broader substrate specificity resulting in vacuolar sequestration (MacDiarmid, Gaither et al. 2000, Li, Bagley et al. 2008, Wang, Okonkwo et al. 2013, Zhang and Kebaara 2022). Furthermore, *ZRC1* and *COT1* encode vacuolar zinc importers that sequester excesses zinc to the vacuole (MacDiarmid, Gaither et al. 2000). *COT1* in particular is dependent on Aft1p expression (Philpott and Protchenko 2008), which suggests increased vacuolar sequestration of zinc. Further studies will be needed to determine the relative contributions of specific zinc transporters and low-affinity multi-metal transporters to copper-zinc-iron homeostatic crosstalk during iron deficiency.

Identification of NMD substrates that are part of the ironome included members of the iron regulon and transporters, metalloreductases, and regulators of iron-sulfur cluster assembly (Fig. 3). This suggests that NMD plays a significant role in iron uptake and distribution within the cell. Examination of the transcriptional activators of the iron regulon and downstream FREs yielded differences in transcript sensitivity to NMD (Fig. 4, and 5). Initially, we grew our cells to mid-log phase in low iron media before RNA analysis. Resulting northern blot analysis demonstrated that *CYH2* pre-mRNA and mRNA levels were identical to when yeast cells were grown under CM.

For physiological relevance we subsequently grew the yeast cells to mid-log phase in CM before exposing them to low iron conditions. Both experimental procedures suggest that NMD modulation of gene expression responds to external stimuli and stressors, enabling adaptation of the organism by adjusting transcript levels to evade NMD when needed. Furthermore, this study demonstrates that changing growth conditions uncover distinct mRNAs that are differentially regulated by NMD, which can be further temporally vary based on the duration of iron starvation events. NMD target transcripts escape NMD in conditions where expression of the protein is required, like low iron in this case.

Transcripts that escape NMD may be attributed to *RLI1*, or *ABCE1* in humans, an essential iron-sulfur cluster protein that is required for ribosome recycling (Zhu, Zhang et al. 2020). Disruption of iron homeostasis affects mitochondrial Fe-S synthesis, which impairs the functionality of Rli1p and abrogates NMD activity (Zhu, Zhang et al. 2020), providing a possible explanation of how some transcripts escape regulation. Related to Rli1p, *LTO1* was found to be upregulated in NMD mutants in normal growth conditions (Zhang, Olaniyan et al. 2025). *LTO1* encodes a substrate-specific adaptor protein that works in conjunction with Yae1p for the specific assembly of Rli1p (Paul, Mühlenhoff et al.

2015). Lto1p is required for the insertion of the iron–sulfur cluster into the Rli1p apo-protein, a critical step in generating mature, functional Rli1p (Lindahl 2019). Since Rli1p is essential for ribosome recycling, proper Fe–S cluster insertion by Lto1p is necessary to maintain efficient dissociation of post-termination ribosomal complexes and overall translational capacity. Perturbation of Lto1p activity could therefore compromise translation and, by extension, NMD efficiency, which is inherently translation dependent. In normal iron levels, *LTO1* transcript levels are elevated in NMD-deficient cells, indicative of potential targeting by the NMD pathway. This model suggests a mechanistic link in which Fe–S cluster metabolism supports ribosome biogenesis and recycling, thereby sustaining NMD activity. In turn, NMD regulates *LTO1* transcripts, creating a feedback loop.

Finally, stabilization of mRNA transcripts may reside in the nature of the mRNAs themselves. Notably, while different mRNA isoforms can be produced under different cellular conditions, approximately five percent of the *S. cerevisiae* genome contains introns (Edwards and Johnson 2019). None of the transcripts examined here contain introns except for our NMD control, *CYH2* (Fig. 4). The different mRNA isoforms we observed could be attributed to alternative transcription start sites or alternative polyadenylation (APA) resulting in transcripts with varying 3'UTR lengths, and condition-specific sequence elements that mediate transcript stability and mRNA turnover (Geisberg, Moqtaderi et al. 2023). Such fine-tuned control is of particular importance regarding iron homeostasis, as fast mRNA regulation would allow for quicker adaptation to fluctuations in iron availability. Future studies that evaluate mRNA isoforms of iron regulon genes in conjunction with the sequence and structure of the individual mRNAs as the cell responds to iron starvation will be clarifying next steps as to how iron availability influences post-transcriptional regulation of iron homeostasis by NMD on a transcript-specific level. Evaluation of corresponding protein levels would also be a valuable next step in the determination of physiological consequences of this regulatory crosstalk under iron scarcity, and whether NMD-mediated changes in iron regulon transcript abundance are ultimately reflected in protein abundance and the cell’s ability to adapt to changes in iron availability.

### Declarations

## Funding

This work was supported by the National Institute of General Medical Sciences of the NIH under Award Number R15GM117524. The content is solely the responsibility of the authors and does not necessarily represent the official views of the National Institutes of Health.

## Author Contribution

J.C, J.C, and B.K, conceived and designed the experiments. J.C, J.C, and B.K, acquired the data. J.C, J.C, and B.K, analyzed and interpreted the data. J.C, J.C, and B.K, , contributed to the preparation of the manuscript and approved the final version. B.K acquired funding for the research.

## Acknowledgement

We thank Dr. Javier Seravalli from University of Nebraska Spectroscopy Core Facility for ICP-MS analysis. We also thank the Molecular Biology Core (MBC) facility at Baylor University for equipment and supplies.

## Figures

Fig 1

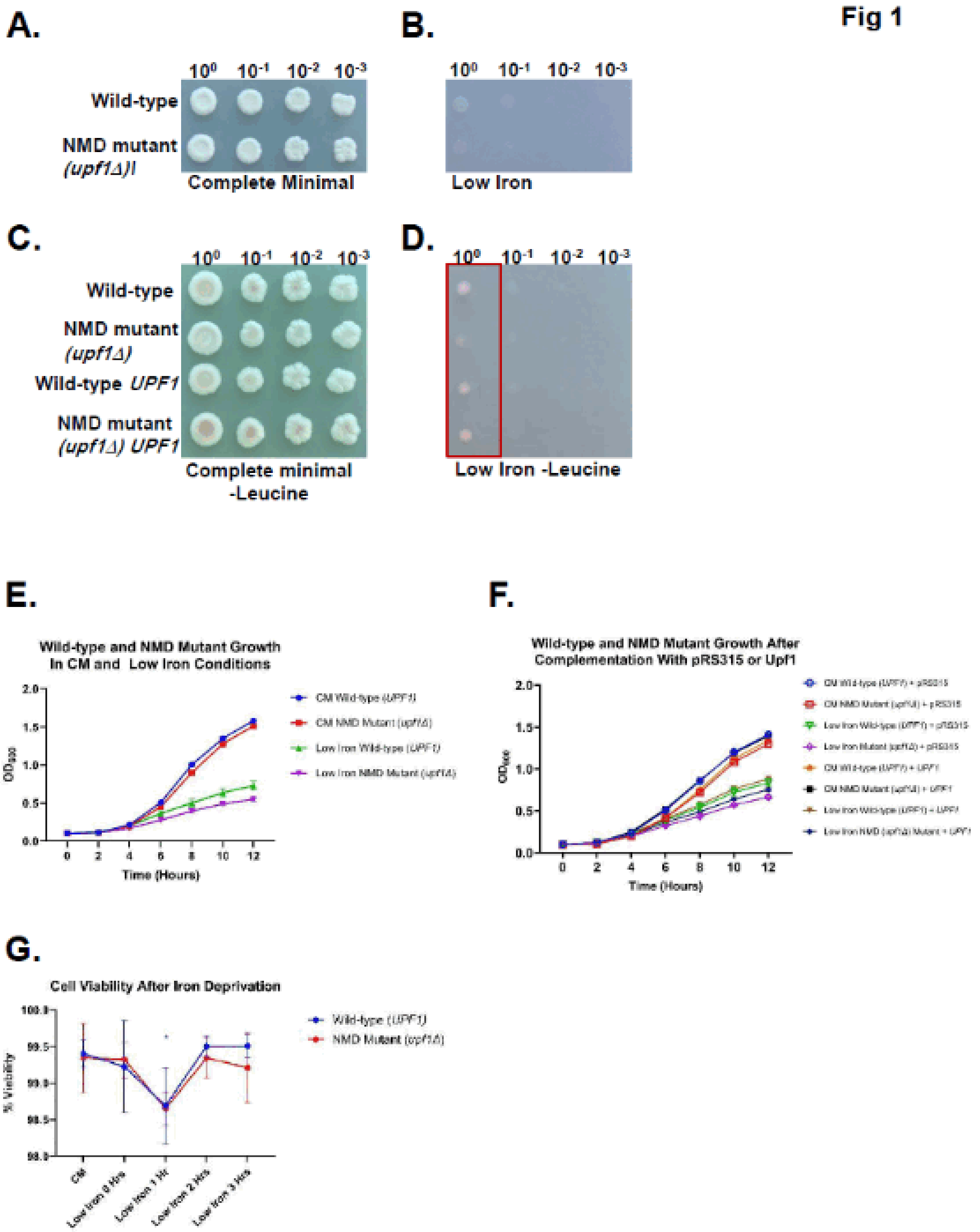

### Figure 1

***The impaired growth of NMD mutant (upf1D) yeast strains in low iron (CM + 100 μM BPS) conditions is complemented by a functional Upf1p.*** A, B) Wild-type and NMD mutant yeast strains were grown to an $OD_{600}$ of 0.4-0.6 before being serially diluted and spotted onto complete minimal (A) and low iron (B) agar plates. Wild-type and NMD mutant yeast strains were each complemented with pRS315 (Leucine vector) and pRS315UPF1 (Leucine vector with *UPF1*) before being grown to an $OD_{600}$ of 0.4-0.6, serially diluted, and spotted onto complete minimal -leucine (C) and low iron -Leucine (D) agar plates. All plates were incubated at 30°C for six days. E) Wild-type *(UPF1*) and NMD mutant *(upf1D)* yeast strains were grown to saturation overnight before rinsing twice with deionized water and subsequently diluted to an $OD_{600}$ of 0.1 before resuspended in complete minimal or low iron media. Growth was monitored every two hours for twelve hours and conducted in triplicate. F) Wild-type and NMD mutant *(upf1D)* yeast cells were complemented with the leucine vector (pRS315) and with a functional Upf1p (pRS315-UPF1) (F) as described above in C. Subsequently, growth was monitored as described in 1E. G) Cell viability of wild-type and NMD mutant *(upf1D)* yeast strains were assessed in complete minimal and low iron media at 0, 1, 2, and 3 hours. Viability was determined by methylene blue and expressed as percentage of viable cells. Two-way repeated measures ANOVA with Sidak's multiple comparisons test were conducted to assess significant effects. Time in low iron conditions was shown to have a significant effect ($p = 0.007$), while genotype and time x genotype interaction were not found to be significant. Sidak's multiple comparisons tests found a decrease in viability in wild-type cells between 0 and 1 hour of iron deprivation ($p = 0.041$), indicated by the asterisk

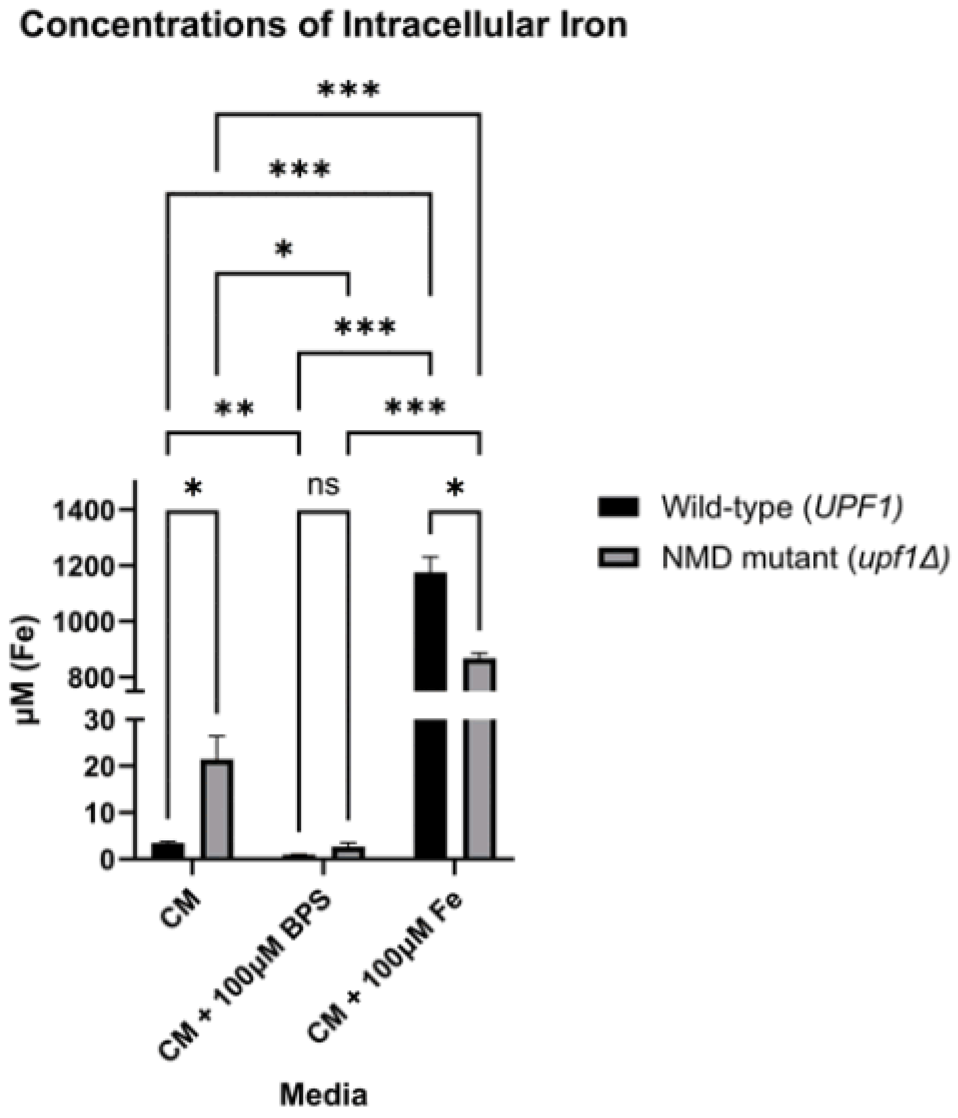

**Figure 2**

***Cellular iron content in wild-type (UPF1) and NMD mutant (upf1D) yeast cells vary depending on iron levels.*** Total intracellular iron content of the wild-type and NMD mutant yeast strains were measured using inductively coupled plasma mass spectrometry (ICP-MS) in normal conditions (CM), low iron conditions (CM + 100 µM BPS), and high iron conditions (CM + 100 µM Fe (III)Cl). The average cellular iron levels are shown with the standard deviations in different growth conditions. Three independent biological replicates were done for each growth condition. Unpaired t-tests with Welch's corrections were conducted to evaluate significance of comparisons. Comparisons between wild-type and NMD mutant cellular iron content in CM ($p = 0.0491$), low iron ($p = 0.0580$), and high iron (0.0170) were conducted, as well as wild-type cellular iron content between growth conditions (CM vs. low iron $p = 0.0063$; CM vs. high iron $p = 0.0008$; low iron vs. high iron $p = 0.0008$), and NMD mutant cellular iron content between growth conditions (CM vs. low iron $p = 0.0211$; CM vs. high iron $p = 0.0002$; low iron vs. high iron $p = 0.0004$).

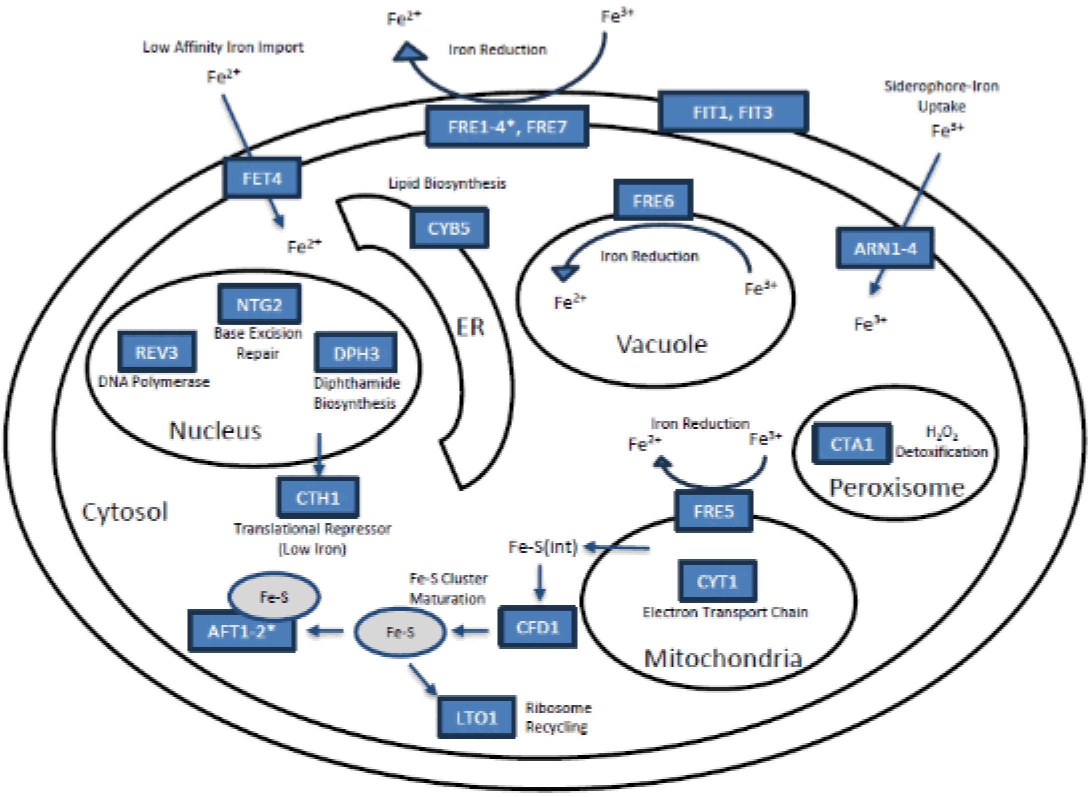

### Figure 3

***Overview of NMD-sensitive yeast ironome transcripts under iron-sufficiency.*** Iron-related proteins encoded by mRNAs demonstrated to be sensitive to NMD are mapped to their primary cellular compartments with associated cellular processes, highlighting the overlap with the iron regulon and illustrating how global iron homeostasis is regulated by NMD. Asterisks denote genes experimentally investigated in this study.

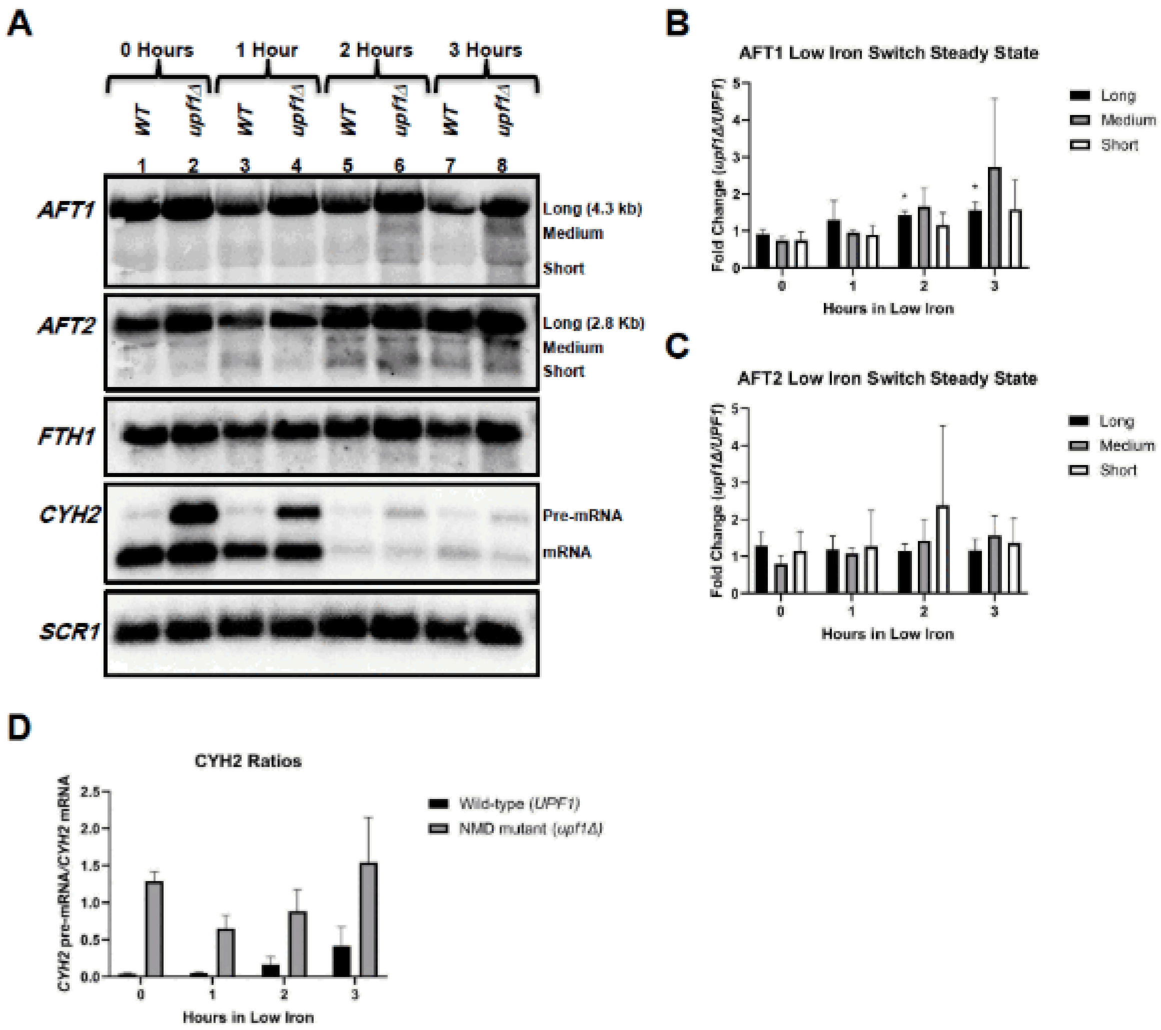

### Figure 4

***mRNA accumulation of AFT1 and AFT2 mRNAs in wild-type (UPF1) and NMD mutant (upf1D) yeast strains over 3-hour adaptation to low iron conditions.*** Wild-type and NMD *(upf1D)* mutant yeast strains were grown to an $OD_{600}$ of 0.4-0.6 before being rinsed twice with deionized water and resuspended in low iron media (100 μM BPS). Cells were harvested at 0, 1, 2, and 3, hours of iron deprivation. The northern blot was sequentially probed with DNA specific to *AFT1*, *AFT2, FTH1, CYH2,* and *SCR1* open reading frames (ORFs). A) A representative northern blot showing *AFT1*, *AFT2*, mRNA transcript accumulation with *FTH1* as a control for conditional regulation in low iron conditions, *CYH2* as control for NMD functionality, and *SCR1* for loading control. B) *AFT1* fold change of each mRNA isoform over 3 hours of iron deprivation (Long ~4.3 kb, Medium ~3.5 kb, Short ~2.2 kb). C) *AFT2* fold change of each mRNA isoform over 3 hours of iron deprivation (Long ~2.8 kb, Medium ~2.1 kb, Short ~1.5 kb). The data in B and C are the mean (± standard deviation) of at least three independent experiments and a t-test was used for significance ($p < 0.05$ = *, $p < 0.01$ = **). D) Quantification of the *CYH2* pre-mRNA to *CYH2* mRNA ratios derived from the controls of triplicate blots.

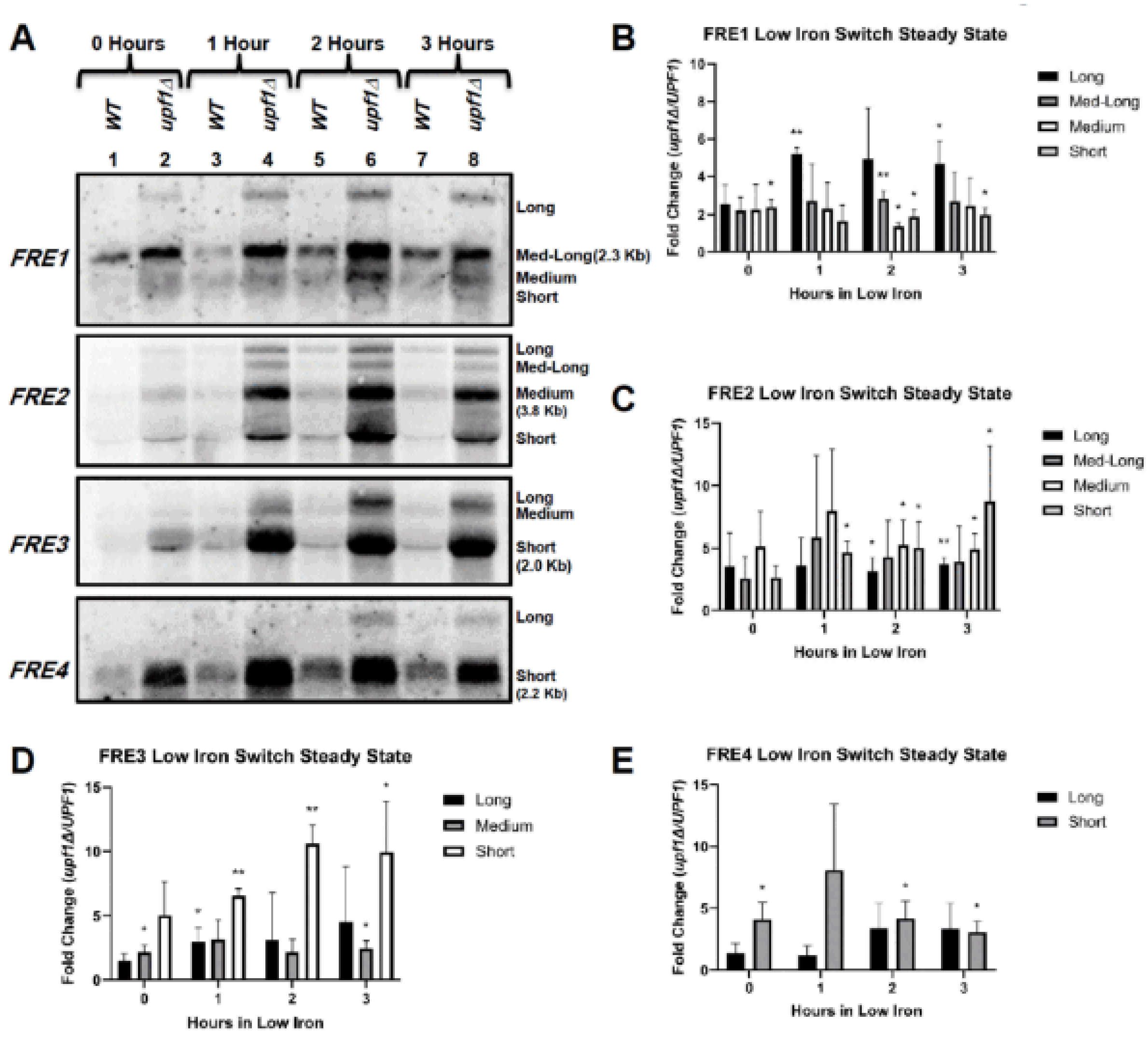

**Figure 5**

***mRNA accumulations of FRE1-4 mRNAs in wild-type (UPF1) and NMD mutant (upf1D) yeast cells over 3-hour adaptation to low iron conditions.*** Wild-type and NMD mutant yeast strains were grown as described in Figure 4 and harvested at 0, 1, 2, and 3, hours of iron deprivation. A) The northern blot was sequentially probed with DNA specific to *FRE1*, *FRE2, FRE3, FRE4, FTH1, CYH2* and *SCR1* open reading frames (ORFs). *FTH1*, *CYH2* and *SCR1* (shown in figure 4) were used as controls as described in figure 4. B)*FRE1* fold change of each isoform over 3 hours of iron deprivation (Long ~6kb, Med-Long ~2.3kb, Short ~2kb). C)*FRE2* fold change of each isoform over 3 hours of iron deprivation (Long ~6.6kb, Med-Long ~5.5kb, Medium ~3.8kb, and Short ~2.5kb). D) *FRE3* fold change of each isoform over 3 hours of iron deprivation (Long ~4kb, Medium ~3.8kb, and Short ~2.5kb). E) *FRE4* fold change of each isoform over 3 hours of iron deprivation (Long ~4kb and Short ~2.2kb). The data in B, C, D and E are the mean (± standard deviation) of at least three independent experiments and t-tested for significance ($p < 0.05$ = *, $p < 0.01$ = **).

### Supplementary Files

This is a list of supplementary files associated with this preprint. Click to download.

- IronNMDSupplementaryTables7.30.20261.docx
- MembraneManuscriptSupplementaryFigures9212026.pptx
